# CA-125 can be part of the tumour evaluation criteria in ovarian cancer trials: experience of the GCIG CALYPSO trial

**DOI:** 10.1038/bjc.2011.593

**Published:** 2012-01-12

**Authors:** J Alexandre, C Brown, D Coeffic, N Raban, J Pfisterer, J Mäenpää, H Chalchal, B Fitzharris, B Volgger, I Vergote, C Pisano, A Ferrero, E Pujade-Lauraine

**Affiliations:** 1 Département d’Oncologie Médicale, Université Paris Descartes, AP-HP, Hôpitaux Universitaires Paris Centre, site Hôtel-Dieu, Paris, France; 2 NHMRC Clinical Trials Centre, Sydney, Australia; 3 Medical Oncology Department, Hôpital des Diaconesses, Paris, France; 4 Department of Medical Oncology, University Hospital of Milétrie, Poitiers, France; 5 Department of Gynecology, Staedtisches Klinikum Solingen, Solingen, Germany; 6 Department of Obstetrics and Gynecology, University of Tampere and Tampere University Hospital, Tampere, Finland; 7 Department of Medicine, University of Saskatchewan. Allan Blair Cancer Centre, Regina, Saskatchewan; 8 Christchurch Hospital, Christchurch, New Zealand; 9 Department of Obstetrics and Gynecology, University Hospital Innsbruck, Innsbruck, Austria; 10 University Hospital Leuven, Leuven Cancer Institute and Department of Gynaecological Oncology, Leuven, Belgium; 11 Division of Medical Oncology, Uro-Gynecological Department, National Cancer Institute, Naples, Italy; 12 Academic Division of Gynecologic Oncology, Mauriziano Hospital, Torino, Italy

**Keywords:** ovarian cancer, CA-125, pegylated liposomal doxorubicin, carboplatin, paclitaxel, radiological

## Abstract

**Background:**

CA-125 as a tumour progression criterion in relapsing ovarian cancer (ROC) trials remains controversial. CALYPSO is a large randomised trial incorporating CA-125 (GCIG criteria) and symptomatic deterioration in addition to Response Evaluation Criteria in Solid Tumours (RECIST) criteria (radiological) to determine progression.

**Methods:**

In all, 976 patients with platinum-sensitive ROC were randomised to carboplatin–paclitaxel (C-P) or carboplatin-pegylated liposomal doxorubicin (C-PLD). CT-scan and CA-125 were performed every 3 months until progression.

**Results:**

In all, 832 patients (85%) progressed, with 60% experiencing a first radiological progression, 10% symptomatic progression, and 28% CA-125 progression without evidence of radiological or symptomatic progression. The benefit of C-PLD *vs* C-P in progression-free survival was not influenced by type of first progression (hazard ratio 0.85 (95% confidence interval (CI): 0.66–1.10) and 0.84 (95% CI: 0.72–0.98) for CA-125 and RECIST, respectively). In patients with CA-125 first progression who subsequently progressed radiologically, a delay of 2.3 months was observed between the two progression types. After CA-125 first progression, median time to new treatment was 2.0 months. In all, 81%of the patients with CA-125 or radiological first progression and 60% with symptomatic first progression received subsequent treatment.

**Conclusion:**

CA-125 and radiological tests performed similarly in determining progression with C-PLD or C-P. Additional follow-up with CA-125 measurements was not associated with overtreatment.

Ovarian cancer ranks as the sixth most common cancer in women worldwide, with developed countries accounting for half of the worldwide burden (Sankaranarayanan and Ferlay, 2006). Advanced ovarian cancer remains a devastating disease, with ∼20% of women having a long-term survival (Hennessy *et al*, 2009).

In recent years, an increased number of conventional cytotoxics and targeted therapies have emerged, with potential activity in ovarian cancer. However, clinical evaluation of these agents in a timely fashion has been problematic, given the limited number of patients, particularly those with recurrent disease. In this context, it is important that the proportion of patients who are excluded from clinical trials because of a lack of measurable disease be minimised. In recurrent ovarian carcinoma (ROC) a significant proportion of patients have only micro-nodular peritoneal carcinomatosis and ascites, which are non-measurable according to Response Evaluation Criteria in Solid Tumours (RECIST) criteria (recently revised, version 1.1) (Therasse *et al*, 2000; Eisenhauer *et al*, 2009). To allow the inclusion of these patients, it was proposed that CA-125 tumour marker be utilised as a tumour progression criterion.

Based on retrospective studies, the Gynecologic Cancer Intergroup (GCIG) proposed that a definition of ovarian cancer progression based on CA-125 doubling be used in clinical trials of first-line therapies (Vergote *et al*, 2000). It was suggested that utilisation of a composite definition of progression based on both RECIST and CA-125 criteria (instead of only one or the other) would increase the statistical power for tests of differences between trial arms regarding progression-free survival (PFS) (Rustin *et al*, 2006). Thus, a public workshop sponsored by the US Food and Drug Administration, American Society of Clinical Oncology, and American Association for Cancer Research (FDA-ASCO-AACR) recommended CA-125 to be used as a surrogate marker of disease progression (Bast *et al*, 2007). They also proposed that CA-125 be included as a part of a composite end point that includes radiological and clinical evaluation. A prospective evaluation to validate CA-125 as a surrogate for disease progression was also recommended. Indeed, some had argued that the use of CA-125 doubling as a progression criterion could alter study results or interfere with patient care by leading to overtreatment (Goonewardene *et al*, 2007).

The CALYPSO phase III trial that compared carboplatin–paclitaxel (C-P) with carboplatin-pegylated liposomal doxorubicin (C-PLD) as treatment for women with platinum-sensitive ROC showed improved PFS times and more favourable toxicity profile with C-PLD (Pujade-Lauraine *et al*, 2010). Sub-studies also demonstrated that C-PLD was associated with a lower incidence of hypersensitivity reactions compared with C-P (Joly *et al*, 2011) and offered a more favourable therapeutic index in patients ⩾70 years old (Kurtz *et al*, 2011). CALYPSO is also the first large randomised trial to incorporate CA-125 (GCIG criteria) and symptomatic deterioration along with RECIST to assess for progression evaluation in women with platinum-sensitive ROC (Pujade-Lauraine *et al*, 2010; Lee *et al*, 2011). These results provide a unique opportunity to specifically address whether CA-125 should be a tumour evaluation criterion in ovarian cancer trials.

## Materials and methods

### Main eligibility criteria of GCIG CALYPSO study

As reported elsewhere, patients with cancer of the ovary or fallopian tube or extra-ovarian papillary serous carcinoma who experienced disease progression longer than 6 months after first- or second-line platinum-based chemotherapy regimen were included (Pujade-Lauraine *et al*, 2010). Patients had to have measurable disease according to RECIST or CA-125 assessable disease according to GCIG criteria or have a histological proven diagnosis of relapse (Therasse *et al*, 2000; Vergote *et al*, 2000).

### Study design

The CALYPSO randomised phase III trial compared the combination of pegylated doxorubicin 30 mg m^−2^ and carboplatin (C) AUC 5, both administered every 4 weeks for six cycles, with paclitaxel (P) 175 mg m^−2^ and C AUC 5 every 3 weeks for six cycles. It was designed as a two-arm parallel non-inferiority trial to determine whether the combination of C-PLD was non-inferior to the standard regimen of C-P. The primary end point was PFS.

### Patient assessment

Clinical examination and CA-125 assessment were required at baseline, then every 3 months until 2 years after treatment discontinuation, and at investigator discretion every 6 months thereafter for 5 years. Imaging (specific X-ray or CT-scan, or ultrasound or MRI) was mandatory at baseline, every 3 months while on treatment and when required during follow-up according to the centre policy and clinical indication. In the initial analysis of the entire population, the diagnosis of progression was based on the occurrence of one of the following events: (1) RECIST progression: occurrence of any new lesion or increase in measurable and/or non-measurable tumour assessed by imagery or clinically, and defined by RECIST 1.0 criteria (Therasse *et al*, 2000); (2) biological progression: CA-125 elevation defined by GCIG criteria (Vergote, 2000); or (3) symptomatic progression or health-status deterioration, including symptomatic deterioration attributable to disease requiring a change in therapy without objective evidence of progression. Patients with increased pre-treatment CA-125 concentrations, which later normalised, or those with pre-treatment CA-125 concentrations in the normal range needed to show evidence of a CA-125 concentration ⩾2 times the upper normal limit on two occasions at least 1 week apart. Patients with increased pre-treatment CA-125 concentrations, which never normalised, needed to show evidence of CA-125 concentrations ⩾2 times the nadir value on two occasions at least 1 week apart.

In the present analysis, the progression type (RECIST, CA125, or symptomatic) that occurred first was identified for each patient. Patients who progressed according to RECIST criteria within 7 days following symptomatic or CA-125 progressive disease (PD) were considered to have RECIST first PD. Similarly, patients who had symptomatic PD within 3 days following CA-125 PD without RECIST PD were considered to have symptomatic first PD. Patients for whom RECIST progression was assessed only on clinical tumour target (e.g., supra-clavicular lymph node) were considered to have symptomatic PD.

### Statistical analysis

PFS was summarised by cumulative incidence curves and compared in each treatment group using multiple recurrent event Cox regression models. Hazard ratios together with 95% confidence intervals (CIs) were calculated. The time to RECIST or symptomatic progression following CA-125 elevation was evaluated by censoring patients at the date of first new treatment or date last known alive. Time to post-study treatment following first progression was evaluated by censoring patients at the date of death or date last known alive.

## Results

In all, 976 patients with platinum-sensitive ROC were randomised. One patient was ineligible because of no ovarian cancer and was excluded from all analyses. After a median follow-up of 22 months, 832 (85%) patients had disease progression at the time of the analysis.

### Type of first progression

In the majority of patients (502 out of 832, 60%), the diagnosis of tumour progression was primarily based on a radiological worsening according to RECIST criteria (Table 1). CA-125 and/or symptomatic progression was also observed within 7 days before or after RECIST progression in 217 (44%) and 51 (10%) patients, respectively. A CA-125 progression without any evidence of RECIST or symptomatic progression was observed in 28% of patients (Table 1). In these patients, the median time from CA-125 elevation to RECIST or symptomatic progression was 2.3 months (C-P, 95% CI: 1.6–2.9) and 2.1 months (C-PLD, 95% CI: 1.4–3.0).

**Figure 1:**
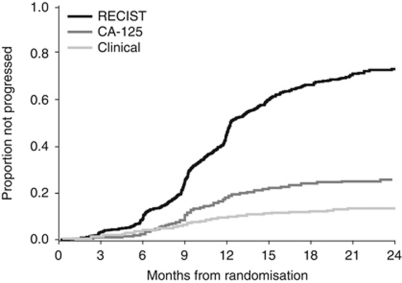
Cumulative incidence of type of progression.

The cumulative incidence of the three types of disease progression is represented in Figure 1. An early progression defined as a progression occurring within 6 months following study inclusion was observed in 146 patients (Table 1). In this subset of patients compared with patients who progressed later, asymptomatic CA-125 elevation as first progression was less common as opposed to symptomatic progression.

### Baseline parameters associated with type of first progression

Radiological first progression was more frequent in patients with measurable disease at baseline compared with those without measurable disease at baseline (Table 2). However, even in patients without baseline measurable disease, radiological progression was the most common type of first progression. In patients who were not assessable for CA-125 at baseline, isolated CA-125 progression occurred rarely (18%). The proportions of each type of first progression were similar between the two treatment arms. Symptomatic progression was more frequent than in patients assessable for CA-125 at baseline (17% *vs* 10% Table 2). Symptomatic first progression was more frequent in patients with mucinous subtype (24%, 95% CI: 10–48) than in other cell types (12%, 95% CI: 10–14; Table 2). Tumour grade and initial tumour stage were not associated with the type of first progression (data not shown).

### Influence of type of first progression on treatment effects

**Figure 2:**
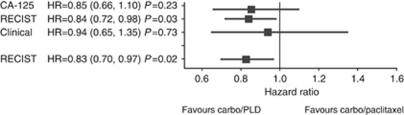
Differences between C-PLD and C-P arms according to type of first progression. ^*^Indicates time to radiological progression post-CA-125 elevation including time to RECIST failure for those patients without prior CA-125 elevation. Abbreviations: C-P=carboplatin–paclitaxel; C-PLD=carboplatin-pegylated liposomal doxorubicin; RECIST=response evaluation criteria in solid tumours.

In the overall study population, C-PLD was associated with improved PFS compared with C-P (11.3 *vs* 9.4 months, *P*=0.005). Figure 2 shows that the benefit of C-PLD over C-P was not influenced by the type of first progression. The median time from isolated CA-125 progression to RECIST progression was similar between C-P (2.3 months) and C-PLD (2.1 months).

### Type of first progression and post-study therapy

The majority (81%) of patients with CA-125 or RECIST first progression received a subsequent treatment; a different treatment strategy was observed following symptomatic progression (Table 3). The median time from progression to post-study therapy was higher in patients with CA-125 first progression (60 days) than in patients with RECIST (28 days) or symptomatic progression (32 days).

## Discussion

Herein we report the modalities of disease progression in patients receiving second- or third-line chemotherapy in a prospective clinical trial that included follow-up with both CA-125 measurements and radiological evaluations. We observed that in the majority of patients (60%), the diagnosis of progression was based on radiological worsening (RECIST criteria). Only a third of the patients had isolated asymptomatic CA-125 progression, probably because of a micro-nodular peritoneal carcinomatosis. In these patients, the median time from isolated CA-125 doubling to radiological or symptomatic progression was 2 months. However, the curve of the cumulative incidence of type of progression suggested that isolated CA-125 progression did not occur earlier than radiological and symptomatic progression in the general population and a change in treatment strategy was delayed until this progression was observed.

Our results suggest that in patients receiving second- or third-line therapy for platinum-sensitive ROC, where physical progression is not observed first, the CT-scan subsequently detects tumour progression close to that of from CA-125 measurements. This finding contrasts with previous reports in patients receiving first-line therapy (Goonewardene *et al*, 2007). This discrepancy is probably related to a higher global tumour volume in relapsing patients compared with patients in the first-line treatment setting. In our study, though symptomatic first progressions were rare, they occurred more frequently in the first 6 months following treatment initiation, and were likely related to very aggressive and chemo-resistant disease.

We observed more radiological progressions in patients with measurable disease at baseline compared with those without baseline measurable disease. However, even in this latter category, nearly half of the patients experienced first radiological progression. This finding suggests that patients without measurable disease at baseline could be considered for studies that define tumour progression based only on RECIST criteria.

Patients with symptomatic progression were not treated as frequently as those with CA-125 or RECIST progression. It can be speculated that a certain number of patients at the time of symptomatic progression were considered to be unable to receive chemotherapy either because of decreased performance status related to occlusive disease or because of other causes. These findings corroborate with those of the MRC OV05/EORTC 55955 trial (Rustin *et al*, 2010). In this trial of women with complete response to first-line chemotherapy, 12% of the patients with symptomatic progression in the delayed-chemotherapy arm could not receive a second-line treatment compared with only 4% of patients randomised to the arm that received chemotherapy as soon as CA-125 progression according to GCIG criteria was observed.

CA-125 measurements and CT-scans performed similarly in comparing efficacy between the two treatment arms in the CALYPSO study. This finding corroborated a previous retrospective report in the first-line chemotherapy setting for advanced ovarian cancer (Rustin *et al*, 2006). It is important to note that this finding cannot be generalised to all anticancer treatments because some biological therapies could interfere with CA-125 production independent of antitumour effect. On the other hand, some biological agents could delay symptomatic progression in patients with increasing CA-125. Such an effect was recently demonstrated with bevacizumab in the phase III GOG218 study in which the addition of concomitant and maintenance bevacizumab to first-line C-P was associated with prolonged PFS in patients with advanced ovarian cancer (Burger *et al*, 2010). The PFS improvement related to bevacizumab was higher when only radiological and symptomatic progressions were taken into account, compared with a composite definition of progression that included CA-125.

In addition, these observations in the CALYPSO trial may not reflect what may be observed in other situations (i.e., in routine practise or trials based on RECIST progression alone). In routine practise and in the CALYPSO trial, patients are often followed with regular physical exams and CA-125 measurements, without regular CT-scans every 3 months. In many registration trials, progression is based only on follow-up with regular CT-scans, and imaging studies are not triggered by CA 125 rise, which could have occurred in the CALYSPO trial. These variations in the follow-up methods could potentially alter some of the conclusions in these specific situations.

In the present study, we provide support to assess the risk of measurement bias in the evaluation of PFS in an open-labeled study such as CALYPSO. Indeed, if the investigators had favoured the experimental arm (C-PLD) and rapidly made a diagnosis of clinical or radiological progression for patients in the control arm (C-P), a higher proportion of non-CA125 first progression in this arm would be expected. However, the current study shows that this was not the case, making the hypothesis of such bias unlikely.

As the management of asymptomatic patients with a rising CA-125 concentration is challenging, it is important to note that follow-up with CA-125 in addition to radiological and physical exams was not associated with overtreatment in our study. In patients with isolated CA-125 elevation, initiation of a new treatment occurred over the longer range of time, that is, 2 months. These results suggest that most clinicians initiated salvage treatment at the time of radiological or symptomatic progression.

In conclusion, our findings support the use of CA-125 measurements, together with radiological and clinical findings, as a criterion for tumour progression for platinum-sensitive ROC. We propose that future clinical trials examining new treatments for ROC continue to incorporate biological assessments in determining disease progression.

## Figures and Tables

**Table 1:** Type of first progression

	**Number of patients (%)**		
**Type of first progression**	**Early progression (*n*=146)**	**Other progressions (*n*=686)**	**All progressions (*n*=832)**
RECIST^a^	88 (61)	414 (60)	502 (60)
Isolated CA-125 doubling (GCIG)	28 (19)	204 (30)	232 (28)
Symptomatic^b^	29 (19)	54 (8)	83 (10)
Unspecified/other^c^	1 (1)	14 (2)	15 (2)

Abbreviations: GCIG=Gynaecologic Cancer Intergroup; RECIST=Response Evaluation Criteria in Solid Tumours.

a Including patients who experienced CA-125 or clinical progression within 7 days before or after RECIST progression.

b Including patients who experienced biological CA-125 within 3 days before or after clinical progression.

c Including tumour progression observed during surgical procedure.

**Table 2:** Baseline parameters associated with RECIST, CA125, or symptomatic progression (PD)

	**Type of first PD**		
	**RECIST (%)**	**CA-125 (%)**	**Symptomatic (%)**
Proportion of progressions^a^	60	28	10
			
*Histological subtype*			
Mucinous (*n*=14)	50	21	14
Other (*n*=818)	61	29	12
			
*Measurable disease (TL)*			
Yes (*n*=551)	68	22	9
No (*n*=281)	46	39	12
			
*Assessable by CA-125*			
Yes (*n*=587)	58	32	13
No (*n*=243)	65	18	9
			
*Treatment arm*			
C-P (*n*=443)	58	28	10
C-PLD (*n*=389)	63	27	9

Abbreviations: C-P=carboplatin–paclitaxel; C-PLD=carboplatin-pegylated liposomal doxorubicin; PD=progressive disease; RECIST=Response Evaluation Criteria in Solid Tumours.

a The totals of the values do not equal 100% because progression of unspecified type and other progressions (*n*=15) were not shown, patients not progressed excluded.

**Table 3:** Type of first progression and post-study therapy

	**Type of first progression**		
	**RECIST**	**CA-125**	**Symptomatic**
Total number of progressions	502	232	83
Number of patients receiving new treatment after progression *n*, (%)	406 (81)	189 (81)	50 (60)
Median time from first progression to new treatment (days)	28	60	32

Abbreviation: RECIST=Response Evaluation Criteria in Solid Tumours.
